# Perceptual and featural measures of mandarin consonant similarity: Confusion matrices and phonological features dataset

**DOI:** 10.1016/j.dib.2023.109868

**Published:** 2023-11-27

**Authors:** Xiaoyu Yu, Youngah Do

**Affiliations:** Department of Linguistics, The University of Hong Kong, Hong Kong SAR

**Keywords:** Confusion matrix, Perceptual distance, Phonological feature, Mandarin Chinese, Consonant similarity

## Abstract

This article presents a comprehensive dataset containing two types of similarity measures for 23 Mandarin consonant phonemes: perceptual and featural measures. The perceptual measures are derived from confusion matrices obtained through native speakers’ identification tasks in quiet and noise-masked conditions. Specific perceptual measures, including confusion rate and perceptual distance, are calculated based on these matrices. Additionally, a phonological feature system is proposed to evaluate the featural differences between each pair of consonants, providing insights into phonological similarity. The dataset reveals a significant positive correlation between the perceptual and featural measures of similarity. Furthermore, distance matrices are generated using the perceptual distance data, and a hierarchical cluster dendrogram is plotted using the unweighted pair group method with arithmetic mean (UPGMA). The dendrogram shows five major clusters of consonants. Future studies can refer to this dataset for quantified perceptual measures of Mandarin consonant similarity. This dataset can also be valuable for future research exploring consonant similarity in perceptual and phonological domains, as well as investigating the influence of linguistic and extralinguistic factors on consonant perception.

Specifications Table

**Table:** 

Subject	Linguistics
Specific subject area	Phonology, speech perception
Data format	Raw, Analyzed
Type of data	Table, Matrix and Spreadsheet (CSV file), Figure (PDF file), Audio (WAV file)
Data collection	Perceptual confusion data of 23 of Mandarin consonant phonemes were obtained from online identification tasks involving 47 native speakers of Mandarin. The audio stimuli used in the identification experiment were generated using a text-to-speech synthesizer. The raw confusion data serve as the basis for calculating the perceptual measures and relevant analysis. The feature system was adapted from a common phonological feature chart. The demographic and linguistic backgrounds of the participants were collected through an online survey before the identification experiment.
Data source location	Institution: The University of Hong Kong City/Town/Region: Hong Kong SAR, PR China
Data accessibility	Repository name: Open Science Framework (OSF) Data identification number: DOI: 10.17605/osf.io/rbt8x Direct URL to data: https://osf.io/rbt8x/

## 1 Value of the Data

• The dataset provides confusion matrices of Standard Mandarin Chinese consonant phonemes, replicating the seminal study by Miller and Nicely [1] on English consonants. These matrices provide insights into the perception of Mandarin consonants by native speakers and allow for comparison with other languages.
• Based on the confusion matrices, detailed perceptual measures (i.e., confusion rate and perceptual distance) of Mandarin consonant phonemes were generated. These measures can serve as the foundation for experiments requiring the examination, manipulation, or quantification of perceptual similarity of Mandarin consonants (e.g., designing experiment stimuli, controlling experiment factors, interpreting perception-related data).
• In addition to perception data, the dataset also proposes six phonological features to distinguish the 23 Mandarin consonant phonemes. The featural difference can quantify the similarity/distance of consonants in a phonological dimension. A significant positive correlation between the perceptual and featural measures of consonant similarity is reported. The raw data and statistical coefficient can be reused by researchers to further investigate the relationship between perception and phonological features of consonants. Other features not proposed in the dataset can also be considered in further studies.
• A hierarchical cluster dendrogram of Mandarin consonant phonemes is plotted based on the perceptual distance. The five major clusters are marked in different colors. This dendrogram can benefit researchers who aim to investigate whether perceptually-based groups of consonants align with natural classes characterized with phonological features.
• Additional information in the dataset includes noise conditions of the stimuli, phonological components (i.e., onset, rime, and tone) and lexical statuses of the stimulus syllables, and participants’ demographic and linguistic backgrounds. These data will allow other researchers to examine how consonant perception varies with these variables.
• Audio stimuli used in the identification tasks are available in the dataset. Readers can manipulate the sound files (e.g., adding other types of noises, filters, or modifying the phonetic correlates) to fit their own experiment objectives. The creation of the dataset itself can serve as a template for further studies investigating the perceptual and phonological similarity of speech sounds in other languages.

## 2 Data Description

The dataset comprises 13 CSV files, a figure, and audio files. The CSV files include a series of confusion matrices, spreadsheets of the perceptual and featural measures, the distance matrices, and the stimulus list and raw experiment data of the identification tasks. All the CSV files are encoded as UTF-8, which allows all embedded symbols, especially the international phonetic alphabet (IPA) symbols, to be readable when opened with Excel software. For an overview of the dataset, please refer to Table 1. The files in the dataset are named with an index at the beginning, and the indices are used to reference specific data items in the following description.

**Table 1:** Overview of the dataset contents.

Index	Format	Description
0101	CSV	A full confusion matrix (Table 2)
0102	CSV	A subdivision of the confusion matrix for the “quiet” condition
0103	CSV	A subdivision of the confusion matrix for the “snr12” condition
0104	CSV	A subdivision of the confusion matrix for the “snr6” condition
0105	CSV	A subdivision of the confusion matrix for the stimuli with the rime /a/
0106	CSV	A subdivision of the confusion matrix for the stimuli with the rime /an/
0107	CSV	A subdivision of the confusion matrix for the stimuli with the rime /ɑŋ/
02	CSV	Phonological features of Mandarin consonant phonemes
03	CSV	The detailed perceptual and featural measures
04	CSV	A distance matrix generated from the raw perceptual distance
05	CSV	A Euclidean distance matrix converted from the raw perceptual distance matrix
06	PDF	A UPGMA dendrogram based on the Euclidean distance matrix (Fig. 1)
07	CSV	The information of the stimulus syllables used in the identification experiment
08	CSV	The raw data from the identification experiment
09	PDF	The content of the survey to collect participants’ demographic and linguistic backgrounds (Chinese version with English translation)
10	WAV	A folder containing all the audio stimuli used in the identification experiment

### 2.1 The series of confusion matrices (CSV files 0101 to 0107)

Table 2 displays the complete confusion matrix of all 23 Mandarin consonant phonemes (as the syllable onsets) across all conditions. The row names in the first column to the left correspond to the target consonants that was actually embedded in the stimuli for each trial. The consonants the participants chose as responses to the target stimuli are listed as the column names on the first row on the top. The number in each cell indicates the frequency that the target-response pair was observed. The column labeled “sum” (second from the right) is the total frequency that each target consonant was identified. Please note that the row sums range between 1126 between 1128, because a minor number of trials (7 trials from 5 participants: quiet_03 *n* = 1; quiet_10 *n* = 1; snr12_04 *n* = 1; snr12_10 *n* = 1; snr6_13 *n* = 3) failed to be recorded due to technical issues of the experiment platform. Hence, we later apply normalization to tackle this discrepancy when calculating the perceptual measures (see Section 2.3). The frequency of correct identifications for each consonant can be obtained along the diagonal of the matrix (e.g., *b* /p/ was correctly identified as *b* /p/ for 787 times). The consonant phonemes in the row and column names are represented in pinyin, a standardized romanization of Mandarin Chinese used in China. In all matrices and spreadsheets, the consonant phonemes are sorted by the pinyin order of onsets (i.e., *b* /p/, *p* /pʰ/, *m* /m/, *f* /f/, *d* /t/, *t* /tʰ/, *n* /n/, *l* /l/, *g* /k/, *k* /kʰ/, *h* /x/, *j* /tɕ/, *q* /tɕʰ/, *x* /ɕ/, *zh* /ʈʂ/, *ch* /ʈʂʰ/, *sh* /ʂ/, *r* /ɻ/, *z* /ts/, *c* /tsʰ/, *s* /s/, *y* /j/, *w* /w/). The corresponding IPA symbols are presented on the other sides of the matrix, i.e., bottom and rightmost edges. This full matrix can also be found in CSV file 01 in the repository [2].

**Table 2:** Full confusion matrix of 23 Mandarin consonant phonemes.

	**b**	**p**	**m**	**f**	**d**	**t**	**n**	**l**	**g**	**k**	**h**	**j**	**q**	**x**	**zh**	**ch**	**sh**	**r**	**z**	**c**	**s**	**y**	**w**	**sum**	
**b**	787	8	3	240	61	2	0	2	15	2	0	0	0	0	0	1	1	1	2	0	1	0	2	1128	**/p/**
**p**	0	714	0	2	0	239	2	0	1	31	45	0	3	0	0	17	1	1	1	68	0	2	1	1128	**/pʰ/**
**m**	0	0	921	1	0	2	153	26	0	1	2	2	1	0	1	2	0	2	0	3	0	0	10	1127	**/m/**
**f**	39	2	2	992	14	2	0	2	2	1	1	0	1	1	0	2	1	1	16	0	45	1	2	1127	**/f/**
**d**	63	3	1	42	779	9	1	2	164	1	2	1	1	0	20	3	3	10	17	1	3	1	1	1128	**/t/**
**t**	0	169	1	3	1	820	2	0	2	30	11	0	3	0	0	25	0	1	1	55	2	1	1	1128	**/tʰ/**
**n**	0	0	53	2	2	4	968	68	0	1	2	0	0	0	1	1	2	14	3	1	1	3	1	1127	**/n/**
**l**	0	0	3	1	1	0	83	948	0	0	2	4	0	0	1	4	0	68	1	0	1	7	4	1128	**/l/**
**g**	4	0	0	1	15	0	0	1	1029	2	2	1	0	3	38	5	2	6	12	1	4	0	1	1127	**/k/**
**k**	1	18	0	2	0	153	3	0	0	850	11	1	0	1	0	37	1	2	0	46	0	0	0	1126	**/kʰ/**
**h**	0	453	0	1	2	90	1	0	1	38	485	1	0	1	1	14	0	1	0	38	1	0	0	1128	**/x/**
**j**	0	1	0	0	0	0	0	0	0	0	1	1065	7	34	6	3	0	1	0	0	1	8	1	1128	**/tɕ/**
**q**	0	0	0	0	0	0	0	0	0	1	0	10	1090	18	1	3	2	1	1	1	0	0	0	1128	**/tɕʰ/**
**x**	0	0	0	1	0	0	0	0	0	0	0	0	15	1106	0	0	2	0	0	1	2	1	0	1128	**/ɕ/**
**zh**	0	0	0	3	0	0	0	0	2	0	0	2	1	3	1054	3	0	11	48	0	0	0	1	1128	**/ʈʂ/**
**ch**	0	3	1	0	0	4	1	0	0	4	0	0	2	1	2	1058	2	0	2	45	0	3	0	1128	**/ʈʂʰ/**
**sh**	0	0	0	1	1	1	0	0	0	0	1	0	1	3	1	3	1082	2	0	0	30	2	0	1128	**/ʂ/**
**r**	0	1	0	0	2	2	2	2	4	1	0	18	0	6	10	2	0	907	3	0	1	162	5	1128	**/ɻ/**
**z**	0	1	0	11	5	0	2	1	7	1	0	4	1	2	19	1	2	0	1033	5	28	2	2	1127	**/ts/**
**c**	0	3	0	2	0	6	1	0	0	0	0	1	1	0	0	18	0	2	3	1089	2	0	0	1128	**/tsʰ/**
**s**	0	1	0	1	2	0	2	0	0	0	0	0	0	3	2	3	31	1	18	3	1060	1	0	1128	**/s/**
**y**	0	1	1	0	3	0	1	1	2	2	0	42	7	17	0	1	0	64	3	0	0	980	3	1128	**/j/**
**w**	0	1	1	0	12	1	4	0	61	3	5	0	0	1	2	0	0	4	0	0	0	1	1032	1128	**/w/**
	**/p/**	**/pʰ/**	**/m/**	**/f/**	**/t/**	**/tʰ/**	**/n/**	**/l/**	**/k/**	**/kʰ/**	**/x/**	**/tɕ/**	**/tɕʰ/**	**/ɕ/**	**/ʈʂ/**	**/ʈʂʰ/**	**/ʂ/**	**/ɻ/**	**/ts/**	**/tsʰ/**	**/s/**	**/j/**	**/w/**		

The full matrix is further subdivided based on the noise conditions and rimes of the stimulus syllables. The subdivisions of different noise conditions are found in files 0102 (“quiet”), 0103 (“snr12”), and 0104 (“snr6”) (see details of the noise conditions in Section 3.2). The confusions of consonants combined with rimes /a, an, ɑŋ/ are displayed in the matrices 0105, 0106, and 0107, respectively. These sub-matrices follow the same layout as the full matrix and can be interpreted in the same manner. The confusion matrices were created with the function “confusionMatrix” in the package “caret” [3] in R [4]. All seven confusion matrices are stored in the folder named “01_confusion_matrix_series”.

### 2.2 Phonological features of mandarin consonant phonemes (CSV file 02)

Table 3 exhibits six phonological features and the values of 23 Mandarin consonant phonemes. This feature system is adapted from Hayes [5]. Note that the features are not exhaustively listed. We select these features as they suffice to distinguish all 23 Mandarin consonant phonemes when used as the syllable onsets. Five of the features, i.e., [sonorant] (son), [nasal] (nas), [continuant] (cont), [delayed release] (dr), and [spread glottis] (sg) are coded in a binary way, in that “1” stands for the positive value and “0” for the negative value (e.g., *m* /m/ is coded as “1” for [+nasal] and “0” for [-continuant]). The last feature, place of articulation (poa), has five categories, namely, labial, alveolar, velar, palatal, and retroflex. Two adaptions are made from the original chart. Firstly, the place of the dorsal fricative *h* is variable, and may be considered as either velar [x] or glottal [h] [6]. Since its articulation may involve airflow throw an open glottis, with phonetic affinity to a glottal fricative /h/ or aspiration, we assign a positive value to its laryngeal feature as [+spread glottis]. Secondly, the place of articulation of the approximant /w/ is marked as labiovelar, which consists of dual articulators of both labial and velar. A copy of the feature chart can be found in CVS file 02 in the repository [2]. In the CSV file, the features in the column names are presented in their abbreviations.

**Table 3:** Phonological features of Mandarin consonant phonemes.

Pinyin	IPA	[sonorant]	[nasal]	[continuant]	[delayed release]	[spread glottis]	Place of articulation
b	/p/	0	0	0	0	0	labial
p	/pʰ/	0	0	0	0	1	labial
m	/m/	1	1	0	0	0	labial
f	/f/	0	0	1	1	0	labial
d	/t/	0	0	0	0	0	alveolar
t	/tʰ/	0	0	0	0	1	alveolar
n	/n/	1	1	0	0	0	alveolar
l	/l/	1	0	1	0	0	alveolar
g	/k/	0	0	0	0	0	velar
k	/kʰ/	0	0	0	0	1	velar
h	/x/	0	0	1	1	1	velar
j	/tɕ/	0	0	0	1	0	palatal
q	/tɕʰ/	0	0	0	1	1	palatal
x	/ɕ/	0	0	1	1	0	palatal
zh	/ʈʂ/	0	0	0	1	0	retroflex
ch	/ʈʂʰ/	0	0	0	1	1	retroflex
sh	/ʂ/	0	0	1	1	0	retroflex
r	/ɻ/	1	0	1	0	0	retroflex
z	/ts/	0	0	0	1	0	alveolar
c	/tsʰ/	0	0	0	1	1	alveolar
s	/s/	0	0	1	1	0	alveolar
y	/j/	1	0	1	0	0	palatal
w	/w/	1	0	1	0	0	labiovelar

### 2.3 The perceptual and featural measures of the consonants (CSV file 03), and the correlation between the two measures

There are 253 possible combinations of two consonants out of the 23 Mandarin consonant phonemes. Pairs of identical consonants such as *n-n* and *sh-sh* have been excluded. The spreadsheet contains detailed perceptual and featural measures of the similarity of the 253 consonant pairs. In the following columns, the number suffixes “1” and “2” respectively indicate the first and second consonants in each pair.

(1) “pair”, “pair_ipa”: The 253 pairs of Mandarin consonant phonemes in pinyin and IPA, respectively.
(2) “pinyin1/2”, and “ipa1/2: The two consonants in each pair in pinyin and IPA, respectively.
(3) “son1/2”, “nas1/2”, “cont1/2”, “dr1/2”, “sg1/2”, and “poa1/2”: The feature values of the consonants in the pair. The feature names are presented in abbreviations.
(4) “son_diff”, “nas_diff”, “cont_diff”, “dr_diff”, “sg_diff”, “poa_diff”: These columns indicate whether the two consonants differ from each other in terms of specific features. If both consonants have the same value for a specific feature, there is no difference. Otherwise, one difference is counted for that specific feature. Note that the labiovelar approximant /w/ does not have a POA difference from both labial and velar consonants.
(5) “featural_difference”: The sum of all the featural difference(s) of each consonant pair.

Note that for each pair, confusions can occur from both directions. For instance, in the pair *b-f, b* /p/ may be misperceived as *f* /f/ and vice versa. The following columns with the suffix “1” present the confusion data when the first consonant in the pair was the target and the second was the response, and the columns ending in “2” present the confusion from the opposite direction.

(6) “target1/2”, and “response1/2”: These columns specify the direction of the confusion by indicating the target and response.
(7) “target_response_freq1/2”: The frequency that the target-response pair was observed in the specific direction.
(8) “confusion_freq”: The sum of “target_response_freq1” and “target_response_freq2”. This sum represents the total frequency that the two consonants in each pair were confused with each other, accounting for confusions from both directions.
(9) “target_freq1/2”: The total frequency of each consonants in the pair being respectively identified as the target.
(10) “total_target_freq”: The sum of “target_freq1” and “target_freq2”. This is the sum of the frequencies that the two consonants in the pair were respectively identified as a target.

There are two major types of perceptual measures in this dataset: confusion rate and perceptual distance. Both measures are calculated by combining the confusions from both directions in each consonant pair.

(11) “confusion_rate”: The confusion rate is calculated for each consonant pair by dividing the (8) confusion frequency by the (10) total target frequency. This also normalizes the confusion measures as not all consonants were identified for the same times (ranging from 1126 to 1128; see Section 2.1). This calculation results in a continuous measure of perceptual similarity within the range [0, 1). A higher confusion rate indicates a higher degree of perceptual similarity and a smaller perceptual distance of the consonant pair.
(12) “perceptual_distance”: The perceptual distance, on the other hand, should be inversely related with the degree of similarity. Hence, we calculate perceptual distance by subtracting the confusion rate from 1. The perceptual distance is thus a continuous measure within the range (0, 1]. In this sense, a larger perceptual distance between two consonants indicates that they are more distinct in perception.

This spreadsheet mainly provides the perceptual and featural measures of similarity of Mandarin consonants. The former quantifies how Mandarin consonants resemble each other in perception, while the latter reflects similarity in the phonological domain. There is a positive correlation between the perceptual distance and number of featural differences (Kendall's *τ_b_* = 0.335, *p* < 0.001).

### 2.4 Perceptual distance matrices (CSV files 04 and 05)

The perceptual distance data are then utilized to generate a distance matrix (CSV file 04). The row and column names represent the consonants of each pair in pinyin. The matrix exhibits symmetry along the diagonal, as the perceptual distance is calculated by combining confusions of two consonants from both directions. Similar to the confusion matrices, the corresponding IPA symbols of the consonants are displayed on the other sides of the matrix. Subsequently, the raw perceptual distance matrix is converted into Euclidean distance metric (CSV file 05) using the function “dist” in the R basic package “stats” [4]. The Euclidean-transformed perceptual distance matrix maintains the layout of the raw perceptual distance matrix. Although the two matrices use different distance metrics, they are consistent in reflecting the relative relationships of the consonant pairs in terms of perceptual distance/similarity. In other words, consonant pairs with a larger raw perceptual distance always have a larger Euclidean-transformed perceptual distance.

### 2.5 Hierarchical cluster dendrogram of mandarin consonant phonemes (PDF file 06)

**Fig. 1:**
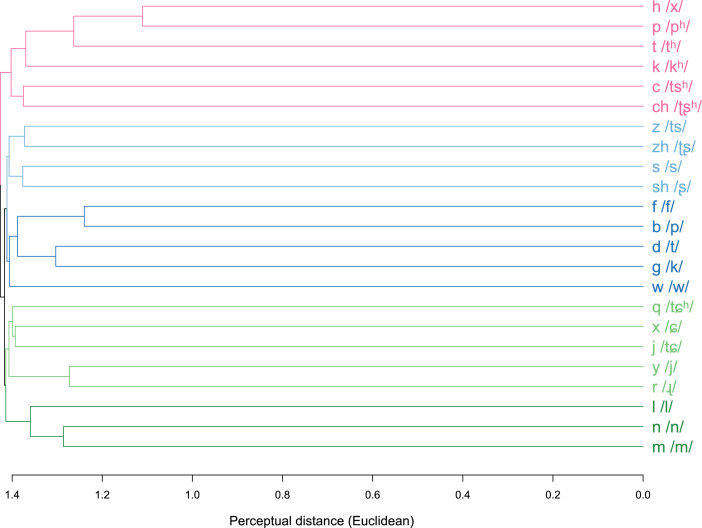
UPMGA dendrogram of 23 Mandarin consonant phonemes based on the Euclidean-transformed perceptual distance. The labels on the tips consist of pinyin symbols of the consonants and the corresponding IPA symbols in slashes.

Fig. 1 is a hierarchical cluster dendrogram of 23 Mandarin consonant phonemes based on the Euclidean-transformed perceptual distance. The dendrogram is generated using the unweighted pair group method with arithmetic mean (UPGMA), implemented with the “upgma” function in the R package “phangorn” [7]. The dendrogram itself is visualized using the package “dendextend” [8]. We adopt the Euclidean distance metric instead of the raw perceptual distance because it enhances the visibility of branches in the dendrogram. However, both distance metrics can produce dendrograms with the same hierarchical structure. Readers are free to try plotting a UPGMA tree with the raw perceptual distance data in the repository [2]. A PDF copy of the dendrogram can be found in file 06.

The horizontal axis of the dendrogram represents the Euclidean distance, ranging from 0 at the tips on the right to a maximum of 1.446 at the root on the left. The distance between two consonants can be determined by locating the point on the horizontal axis where the branches for the two consonants merge. The dendrogram consists of five major clusters, distinguished by different colors marking the branches and tips. Starting from the root on the left side, the tree initially branches into the a smaller pink cluster and a larger cluster. The larger cluster further splits into two secondary clusters. One of the secondary clusters comprises two small clusters in light and dark blue, and the other consists of two small clusters in light and dark green. These clusters group together Mandarin consonants that are similar in perception.

### 2.6 The stimulus list (CSV files 07)

The stimulus list (CSV file 07) shows the details of the syllables used as auditory stimuli in the identification tasks. All the stimuli are monosyllabic.

(1) “stimulus_pinyin_with_tone”, “stimulus_pinyin”, “stimulus_ipa”: The stimulus syllables represented in pinyin with and without tone numbers, and in IPA, respectively. Tones numbers “1”, “2”, “3”, and “4” respectively indicate the four lexical tones of Mandarin, i.e., /55, 35, 214, 51/. These numbers only represent the tone types but not the specific tone values or contours, which are instead marked with the Chao tone letters when necessary. Throughout the dataset and description, the Chao tone letters are only used when IPA transcription of the sounds is necessary, and are always enclosed in slashes.
(2) “onset_pinyin”, “onset_ipa”, “rime_pinyin”, “rime_ipa”, and “tone”: The onsets, rimes and tone numbers of the stimulus syllables.
(3) “lexical”: The lexical statuses of the syllables. The type “common” signifies the presence of at least one common words associated with the syllable. Syllables that are illegal in Mandarin phonotactics are labeled as “systematic gap”, while possible but non-existent syllables are classified as “accidental gap”. Some syllables are identified as extremely “rare” words based on the judgement by the first author, who is a native speaker of Mandarin.

### 2.7 The raw data of the identification experiment (CSV file 08)

The raw experiment data of the identification tasks can be accessed in CSV file 08. The spreadsheet contains the detailed information regarding the participants’ responses in the identification tasks, as well as their demographic and linguistic backgrounds.

(1) “participant”: The participants are identified by anonymous IDs, which consist of the experiment condition they were assigned to, followed by a numeric index. For instance, participant 10 in the snr6 condition is represented as “snr6_10”.
(2) “condition”: The noise conditions the participants were assigned to. The condition “quiet” uses pure audio stimuli without any noise. The “snr12” and “snr6” conditions involve white noise masking the stimuli. For more details about the noise conditions, please refer to Section 3.2.
(3) “trial”: The trial orders of the identification tasks.
(4) “syllable_pinyin” and “syllable_ipa”: The syllables used as stimuli in experiment trials. Both pinyin and IPA transcriptions are provided.
(5) “target_pinyin” and “onset_ipa” The onsets of the stimulus syllables represented in pinyin and IPA. In each trial, the onset consonant is the target for the participants to identify.
(6) “response_pinyin”: The responses chosen by the participants when identifying the target consonants (in pinyin).
(7) “rime_pinyin”, “rime_ipa” and “tone”: The rimes (in pinyin and IPA) and tone numbers of the stimulus syllables.
(8) “lexical”: The lexical statuses of the stimulus syllables (see (3) in Section 2.6).
(9) “accuracy”: This column indicates whether the participant accurately identified the target consonants. If the consonant in the “response_pinyin” column matches that in “target_pinyin”, the trial is counted as “correct”. Otherwise, it is coded as “incorrect”.
(10) “L1”, “dialect1”, and “dialect2”: The linguistic backgrounds of the participants. “L1” stands for the participants’ first languages. The columns “dialect1” and “dialect2” list the Chinese varieties the participants speak in addition to Mandarin, if any.
(11) “gender” and “age”: Demographic information of the participants.

### 2.8 The survey (PDF file 09)

Before the identification experiment, participants’ demographic and linguistic background information was collected via an online survey (see Section 3.3). The content and translation of the survey can be found in PDF file 09.

### 2.9 Audio files of the experiment stimuli (WAV files in folder 10)

The audio files of the stimuli used in the experiment can be found in the folder “10_stimulus_audio”. Three of the subfolders contain the audio files of the target syllables used as stimuli in the formal identification tasks, with the folder names indicating the respective noise conditions (“quiet”, “snr12” and “snr6”). The other subfolder titled “practice” contains the audios of the two syllables used in the practice trials (see Section 3.3). In all folders, the audio files are named with the pinyin symbols indicating the syllable’s segments and tone numbers. For instance, the sound file “san4.wav” in subfolder “snr12” is the syllable /san51/ masked in white noise at 12 dB signal-to-noise ratio (see details about the noise conditions in Section 3.2). Readers are free to use these audio stimuli when replicating or developing the experiment.

## 3 Experimental Design, Materials and Methods

To measure the perceptual distance, we adopted the seminal perception experiment paradigm by Miller and Nicely [1]. This paradigm involves an identification experiment, where participants are required to identify the target sounds they hear in the audio stimuli under quiet or noise-masked conditions. The raw confusion data collected from the identification tasks serve as the basis for generating the confusion matrices and the other perceptual measures.

### 3.1 Participants

We recruited 71 speakers of Standard Mandarin Chinese from our institute in Hong Kong. However, we excluded 24 participants who reported other non-Mandarin varieties of Chinese as their native languages, such as Cantonese. Among the remaining 47 participants, 44 of them are native speakers of Mandarin. Additionally, there are 3 participants whose native language is a Mandarin variety (i.e., Southwestern Mandarin or Sichuanese). Due to the close affinity of this variety with Standard Mandarin Chinese and the fact that these participants use Mandarin as the dominant language in their daily life, we assume that their Mandarin proficiency is near-native and thus have kept their perception data in the dataset. There are 16 participants who also reported fluency in other Chinese varieties, while considering Mandarin as their native and dominant language. All participants claimed proficiency in pinyin. None of the participants reported any hearing impairment. Each participant received 50HKD as a reward of participation.

### 3.2 Stimuli

The target sounds for the identification experiment are the 23 consonant phonemes of Mandarin. These 23 consonants were used as the syllable onsets and concatenated with 3 rimes /a, an, ɑŋ/, yielding a total of 69 syllables. To minimize the coarticulatory effect on the consonants from the vowels, we specifically chose the rimes with only low and unrounded vowels. Note that the Mandarin morpheme *r* involves a free variation between a retroflex approximant [ɻ] and fricative [ʐ] [9,10]. Inspecting the waveforms and spectrograms of the phoneme *r* in our stimuli (computer-generated; see below), we also noticed some minor frication noise (e.g., aperiodic components in the waveform), which could potentially lead to perceptual similarity with consonants involving high frication. However, as previous research reveals that frication noise is not a consistent and crucial acoustic property of *r* [9,10], we treat this consonant phoneme as an approximant ([+sonorant]) and adopt the transcription /ɻ/. For the syllables with the alveolo-palatal consonants (i.e., *j* /tɕ/, *q* /tɕʰ/, and *x* /ɕ/), an extra glide *i* [j] was inserted between the onset and the rime to conform to Mandarin phonotactics. We acknowledge that this will unavoidably increase the perceptual distance of the alveolo-palatal consonants from the other consonants. The only illegal sequence (systematic gap) is /ɻa/, but we still kept this syllable in order to counterbalance the frequencies of all onsets and rimes. The 69 syllables were then combined with the 4 lexical tones of Mandarin (i.e., /55, 35, 214, 51/), resulting in a total of 276 distinctive syllables. Please note that a small number of segment-tone combinations are accidental gaps or have low lexical frequency. Information regarding the phonological components and lexical statuses of the stimulus syllables can be found in the stimulus list in CSV file 07.

All the audio stimuli were generated using *Amazon Polly* [11], an online text-to-speech service, employing a female Mandarin voice, *Zhiyu*, with the neural engine setting. Three noise conditions were created. For the quiet condition (“quiet”), the original audio files were normalized to 70 dB intensity with no added noise. For the two noisy conditions (“snr12” and “snr6”), the sound files were masked with white noise at signal-to-noise ratios (SNR) of 12 dB and 6 dB, respectively. After the noise was added, the overall intensity was also scaled to 70 dB to match that of the quiet condition. All the sound edition was performed in *Praat* [12], and the noise mixing was carried out with the Praat script “Mixing Speech and Noise” developed by McCloy [13].

### 3.3 Procedure

The experiment was developed with *PsychoPy* and performed online via *Pavlovia* [14]. The entire procedure took an average of 45 min. All participants completed the experiment using their personal computers. The experiment was displayed in full screen, and participants were advised to sit in a quiet room and put on their headphones to reduce distractions.

**Fig. 2:**
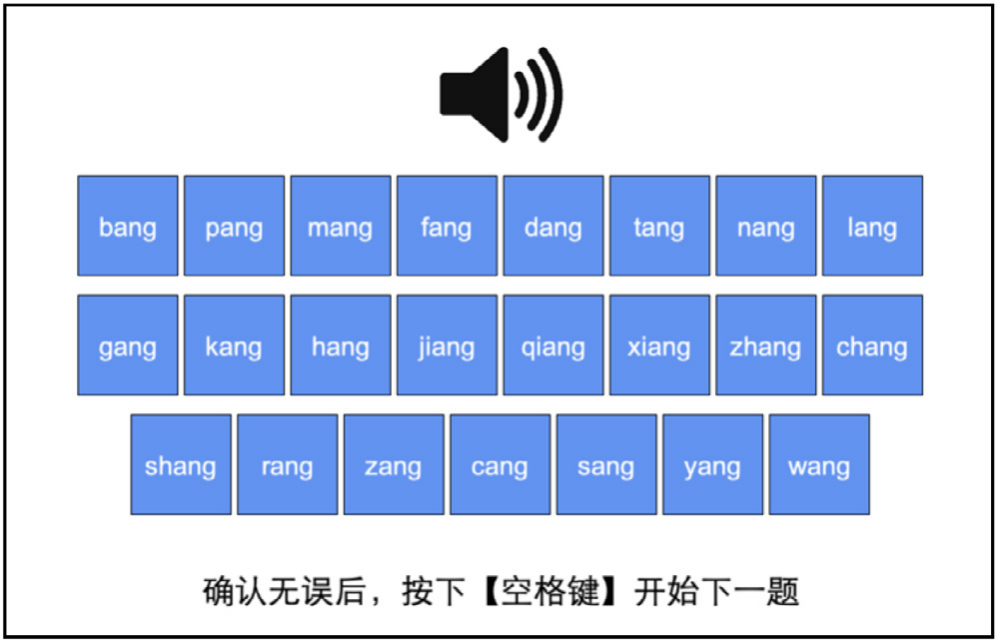
Still from one of the identification trials, in which the participant was presented with 23 choices of syllables in pinyin differing in only the onsets. The caption in Chinese on the bottom translates as “after making your choice, please press the [SPACE] key to continue”.

The demographic information was first collected via *Qualtrics* [15] (see PDF file 09 for the content and translation of the survey). Participants were then randomly assigned to one of the three noise conditions, i.e., “quiet” (*n* = 18), “snr12” (*n* = 14), and “snr6” (*n* = 16). Each trial was preceded by a fixation point at the center of the screen lasting for 0.5 s. Then, one of the 276 syllables was played. After the audio was fully played, 23 buttons showed up on the screen, each containing one syllable transcribed in pinyin, as in Fig. 2. The 23 pinyin syllables all shared the same rime with the stimulus syllable in the audio, but differed only in the onset consonants. The 23 consonants in the buttons followed the pinyin order of onsets, and the order remained in situ across all trials. Tones were not marked in the pinyin syllables. In each trial, participants were instructed to identify the syllable they heard and choose the correct pinyin by clicking the corresponding button with their mouse. Given their self-reported high proficiency in pinyin, we assume that they were fully capable of choosing the pinyin symbols that matched their perception. As the syllables in the buttons only differed in onsets, their choices should reflect their identification of the onsets. They were also instructed to ignore the rimes and tones and were informed that it was possible to hear nonwords (systematic and accidental gaps). They were allowed to change their choice before they finally pressed the space key on the keyboard to move on to the next trial. All instruction was presented in simplified Chinese characters in *Heiti SC* font. Prior to the formal trials, there were two practice trials with stimuli (i.e., /tuŋ55/, /ɕi55/) that were not used in the main rounds. No noise was added to the practice stimuli. This phase served to familiarize the participants with the experiment procedure.

Each participant completed a total of 552 trials, with the 276 syllables repeated twice. The order of the stimulus presentation was randomized across participants. To minimize the potential unpleasantness caused by the masking noise, a music break was inserted every 110 trials, during which relaxing music was played. Participants had the option to proceed by pressing the space key after a 10-second interval or continue listening to the music until the break automatically ended after 60 s. A total of four music breaks were incorporated throughout the experiment. To keep the experiment procedure consistent across all conditions, music breaks were included in both the quiet and noisy conditions.

## Limitations

Not applicable.

## Ethics statement

The experiment above has been approved by the Human Research Ethics Committee of The University of Hong Kong (reference number: EA220424). The performance of the experiment was in accordance with its guidelines and regulations. Participants were asked to give their informed consent by clicking the corresponding button in the online survey.

## Data Availability

Mandarin Consonant Similarity: Perceptual and Featural Measures (Original data) (OSF)
